# Vitamin K2 Enhances Fat Degradation to Improve the Survival of *C. elegans*

**DOI:** 10.3389/fnut.2022.858481

**Published:** 2022-04-15

**Authors:** Zhi Qu, Lu Zhang, Wei Huang, Shanqing Zheng

**Affiliations:** ^1^Medical School, Henan University, Kaifeng, China; ^2^School of Nursing and Health, Henan University, Kaifeng, China; ^3^School of Basic Medical Sciences, Henan University, Kaifeng, China; ^4^Laboratory of Cell Signal Transduction, Henan Provincial Engineering Centre for Tumor Molecular Medicine, Medical School of Henan University, Kaifeng, China

**Keywords:** vitamin K2, *C. elegans*, fatty acid (composition in), β-oxidation degradative pathway, longevity (herd life)

## Abstract

The beneficial effects of vitamin K (VK) on various chronic age-related syndromes have generally been considered dependent on its antioxidant effects. However, due to the distinct bioavailability and biological activities of VKs, exactly which of these activities and by what mechanisms they might act still need to be elucidated. In this study, we found that VK2 can extend the lifespan of *C. elegans* and improve the resistance to pathogen infection, heat stress and H_2_O_2_-induced inner oxidative stress. Importantly, the roles of VK2 on aging and stress resistance were shown to be dependent on enhanced fat metabolism and not due to its antioxidant effects. Moreover, the genes related to fat metabolism that were up-regulated following VK2 treatment play key roles in improving survival. Obesity is a leading risk factor for developing T2DM, and taking VKs has been previously considered to improve the insulin sensitivity associated with obesity and T2DM risk. However, our results showed that VK2 can significantly influence the expression of genes related to fat metabolism, including those that regulate fatty acid elongation, desaturation, and synthesis of fatty acid-CoA. VK2 enhanced the fatty acid β-oxidation activity in peroxisome to degrade and digest fatty acid-CoA. Our study implies that VK2 can enhance fat degradation and digestion to improve survival, supporting the effectiveness of VK2-based medical treatments. VK2 is mainly produced by gut bacteria, suggesting that VK2 might facilitate communication between the gut microbiota and the host intestinal cells to influence fat metabolism.

## Introduction

Metabolic syndrome has become a major health problem affecting people worldwide and has resulted in a huge cost to health care systems with potential economic complications (1). The syndrome includes two major metabolic disorder diseases: obesity and type 2 diabetes mellitus (T2DM). Importantly, obesity is the leading risk factor for the development of T2DM. Research has shown that obesity or a high body mass index (over 35 kg/m^2^) confers a significantly greater risk to the development of diabetes (2–5). The results of clinical and animal studies have supported the idea that the intestinal microbiota might help regulate host obesity (6–9); however, the underlying molecular mechanisms influencing such obesity-related pathways are poorly understood. The intestinal microbiota impact obesity, fatty liver diseases, and weight loss by way of the ability to induce the expression of genes related to lipid metabolism (6, 10). The gut microbiota functionally influences host genetic events mainly through specific media molecules produced by the bacteria (10–13). However, exactly which molecular or metabolic products are the essential transmitters remains to be elucidated. Vitamin K2 (VK2) is commonly produced by gut bacteria and is fat-soluble, allowing it to be easily transferred into the host intestinal cells (14). Recently, *in vitro* studies have shown that VK2 has potential beneficial effects on chronic aging diseases, such as cardiovascular disease (15), Alzheimer's disease (16, 17), cancer (18), osteoporosis (19, 20), and Parkinson's disease (21). VK2 has also been supplied to patients with diabetes and obesity to evaluate its pharmacological activity (22), and the association between VK2 and T2DM, obesity, and fat mass are becoming more clear (23, 24). However, the bioactive effects of VK2 as a potential gut microbiota product and its influence on the health, aging, and fat metabolism of the host have not been extensively studied.

Nematode *Caenorhabditis elegans* (*C. elegans*) is a extensively used as a powerful model organism for physiological, cell division, molecular biology and many other researches (25), and provide a perfect model to study the interaction between intestine and other organic systems (26, 27). In order to test whether VK2 is a potent microbiota product to influence the health of host, we used *C. elegans* to test the biological effects of VK2 on aging and fat metabolism in this study. This work might be helpful in finding the reasons why VK2 plays an important role in ameliorating the outcome of patients with obesity and diabetes mellitus.

This study aimed to test the lifespan and physiological changes, especially fat metabolism, influenced by VK2 treatment in worms, and to explore whether VK2 can affect longevity and age-related metabolism disease through reducing the fat contents in the animal body.

## Materials and Methods

### Strains

Mutant and wild-type *C. elegans* strains used in this study were acquired from the Caenorhabditis Genetics Center (CGC). Worms were cultured using standard methods as previously described (28). Strains were fed with OP50 *Escherichia coli* and cultured at 20°C unless otherwise indicated. Strains used in this study: wild-type: N2, CF1038: *daf-16(mu86)*, AA10: *daf-12(rh286)*, BX160: *fat-7(wa36) fat-5(tm420)*, CB6738: *lys-7(ok1384)*, BX275: *fard-1(wa28)*, RB908: *pmp-1(ok773)*, and RB2102: *hacd-1(ok2776)*.

### Worm RNAi

The designated parts of the target genes were cloned into Vector L4440 and expressed in an RNAi bacterial strain (HT115). A double-stranded target RNAi (vector, L4440) strain was cultured and used to inactivate the target gene function (29). Eggs from worms fed with the target RNAi bacteria were transferred to fresh NGM plates containing the same bacteria and allowed to grow at 15°C for 3 days with or without the presence of VK2. Stage 4 larva were then transferred to the same RNAi NGM plates with or without VK2 to perform the indicated experiments. A total of 1 mM isopropyl-B-D-thiogalactopyranoside (IPTG) was used in the RNAi NGM plates to induce the double-stranded RNA. The L4440 carrying an empty RNAi plasmid was used as a negative control in the RNAi experiments (29).

### Oil Red O Staining

Oil Red O staining is the standard method to test fat content in worms, and performed as previously reported (30–32). Synchronized eggs were cultured on OP50 plates with or without 0.1 μM VK2 treatment for 3 days at 20°C. Worms were then collected and washed in M9 three times and then fixed with the addition of 600 μL of 40% isopropanol to the worm pellet and rocked at room temperature for 3 min. Subsequently, the worms were stained in Oil Red O working solution for 2 h at room temperature with rotating at 30 rpm. After washing, the worms were mounted and imaged with a color camera outfitted with DIC optics. The Oil red O intensity was then quantified. At least 90 animals were imaged in at least three separate experiments using a Zeiss Axioplan microscope (Zeiss). The red intensity was captured and quantified using Image J software (NIH).

### Longevity and Survival

Synchronized young adults were transferred to NGM plates seeded with OP50 and cultured on plates with or without 0.1 μM VK2 at 20°C. The worms were scored every day by counting the number of worms that were moving (alive) until the day that all the worms had died. To compare the survival rates between the different treatments, mean survival rates were calculated using the Kaplan-Meier method, and the significant difference in the overall survival rates was determined using the log-rank test (33, 34).

For the pathogen infection assay, NGM plates with or without 0.1 μM VK2 were seeded with *Pseudomonas aeruginosa* (PA14) and cultured overnight before use. The young adults were then transferred to NGM plates seeded with *Pseudomonas aeruginosa* (PA14) and cultured at 20°C. The worms were assessed every 12 h as dead or alive (33). Worms were considered dead when they failed to respond to a touch with a platinum wire. At least three independent repeat experiments were performed.

For heat tolerance assays, worms at day 5 were transferred to NGM plates with or without 0.1 μM VK2 and incubated at 37°C. The animals were then scored as dead when they did not respond to a gentle touch with a platinum wire and were assessed every 2 h. At least three independent repeat experiments were performed.

For oxidative stress induced by H_2_O_2_, synchronized L1 worms were cultured in M9 with or without 0.1 μM VK2 at 20°C for 3 days. Then, H_2_O_2_ was added into the M9 to a final working concentration of 10 mM. About 50–100 μL M9 with at least 50 worms were selected to be scoured as alive or dead every 2 h. The survival percentages were then calculated. At least three independent repeat experiments were performed.

### RNA-Sequencing and Analysis

Synchronized young adults were cultured on NGM plates with or without 0.1 μM VK2 and incubated at 20°C for 7 days. The worms were transferred to new NGM plates with or without VK2 every day to get rid of the progeny and then collected. Total RNA quality was measured using a NanoDrop ND-1000 (Agilent Technologies). Library preparation was performed using a KAPA Stranded RNA-Seq Library Prep Kit (Illumina), and the quality was confirmed using an Agilent 2100 Bioanalyzer (Agilent Technologies). Transcriptome alignment and quantification were performed using Hisat2 software. *C. elegans* genome version WBcel235 was used as the reference. Differentially expressed genes were identified using the Ballgown package of R project to calculate the Fragments Per Kilobase of gene/transcript model per million mapped fragments (RPKM). The newest version of KEGG pathway enrichment and Gene Ontology were performed using R project or Python developed by Aksomics Inc. The heat maps were generated using Group FPKM (The average of log scaled FPKM: log_2_(FPKM + 1) of the genes in the groups).

### Progeny and Reproductive Activity

The synchronized L1 worms were cultured on plates with or without 0.1 μM VK2 at 20°C for 3 days. A single worm was picked to new plates for each treatment and transferred onto fresh plates, respectively, every day until no eggs were produced. After transferring, the plates containing the eggs yielded by one single worm were put at 25°C for 2 days, and the hatched worms and dead eggs were scored for each plate.

### Real-Time PCR

Synchronized young adult worms were cultured on plates with or without 0.1 μM VK2 at 20°C for 7 days. The worms were then transferred to new NGM plates for each treatment, respectively, every day to remove the progeny. The worms were then collected, and total RNA was extracted using RNAiso Plus (Takara) and converted to cDNA using a High Capacity cDNA Reverse Transcription Kit (Applied Biosystems). The real-time PCR was performed using Power SYBR Green PCR Master Mix (Applied Biosystems) and an ABI 7500 system. The relative expression levels of the genes were assessed using the 2^−ΔΔCT^ method and normalized to the expression of the *cdc-42* gene. *P*-values were calculated using a two-tailed *t*-test (32–34). The primers used are listed in Supplementary Table 1.

## Results

### VK2 Can Improve the Survival and Stress Resistance of *C. elegans*

**Figure 1:**
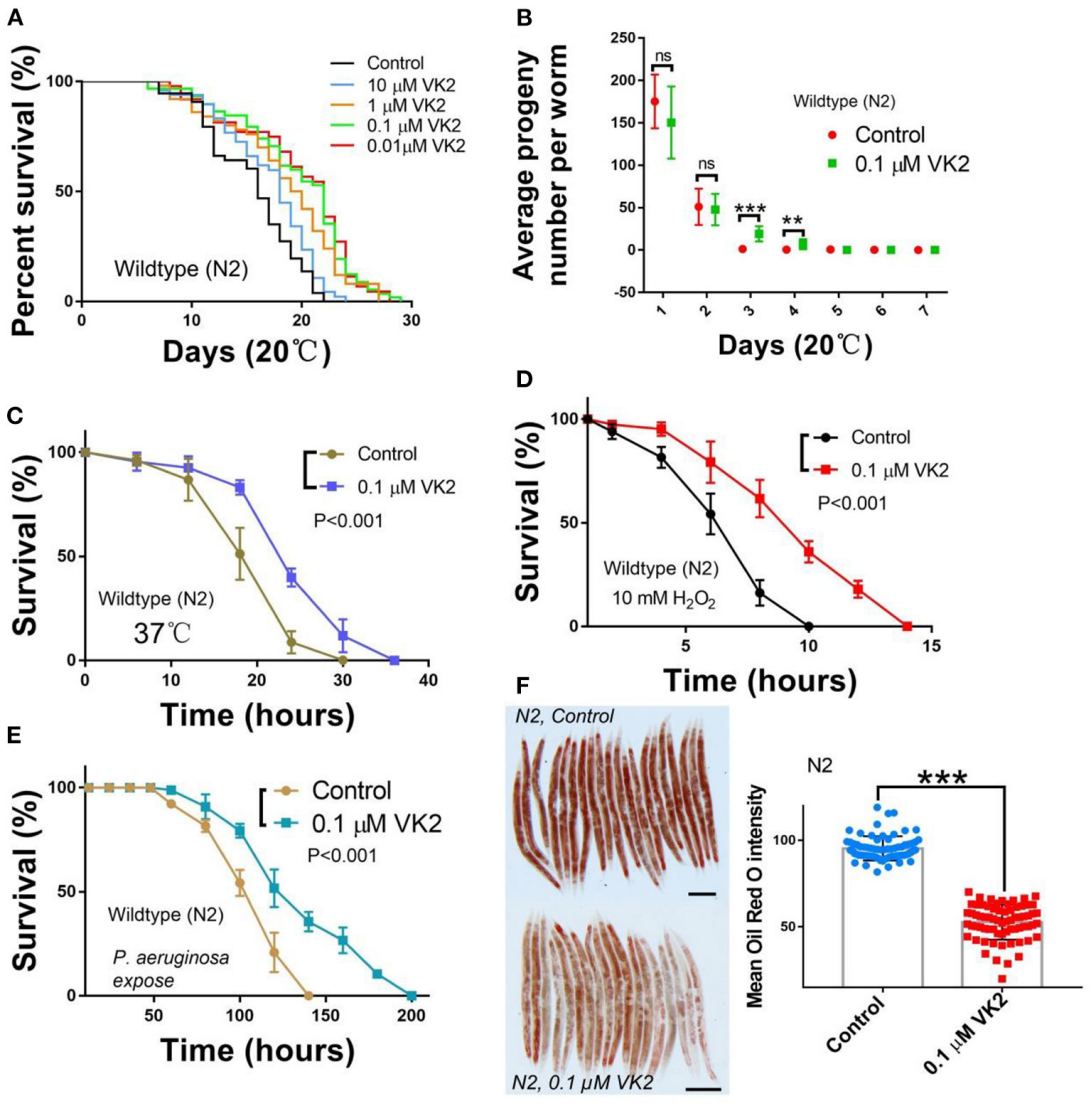
VK2 extends the lifespan of N2 worms and improves survival under stress. **(A)** Wild-type N2 worms were treated with different concentrations of VK2; 0.1 μM VK2-treated worms had the strongest longevity extension. The treatments were repeated at least three times, and the sample size was larger than 60 worms in each treatment. The data are summarized in Table 1. **(B)** 0.1 μM VK2-treated N2 worms had longer productive spans and more progeny at day 3 and day 4. The treatments were repeated at least three times, and the sample size was larger than 20 in each experiment. The survival of N2 worms was improved under heat **(C)**, H_2_O_2_ **(D)**, and *P. aeruginosa* treatments **(E)**. The treatments were repeated at least three times, and the sample size was larger than 60 worms in each experiment. The *P*-values were carried out using a *t*-test. **(F)** VK2-treated N2 worms had a lower fat content compared with the controls. Oil Red O was used to stain the fat droplets in the worm body, and the changes in lipid content were analyzed using Image J software. The treatments were repeated at least three times, and the sample size was larger than 30 in each experiment. Scale bar: 100 μM. ***P < 0.001. VK2, vitamin K2. **P < 0.01.

The role of VK2 on aging-related diseases has been reported in several prior studies (22, 35); however, the effect of VK2 on lifespan has not been addressed. We use *C. elegans* as a model to test the effect of VK2 on lifespan. It was observed that the low-dose VK2 (0.01–10 μM) treatment extended the lifespan of wild-type worms, with the 0.1 μM VK2 treatment having the strongest effect on lifespan extension (Figure 1A), demonstrating about a 20% mean lifespan extension and a maximum lifespan extension from 22 days to 29 days (Table 1). These experiments also showed that VK2 (0.1 μM) could prolong the reproductive span of wild-type worms, with the VK2-treated worms producing more progeny at day 3 and day 4 relative to the control worms (Figure 1B).

**Table 1:** Lifespan of worms treated with VK2.

**Strains**		**Mean life span ±SEM (days)**	**Maximum life span (days)**	***P*** **-value**	***N***
*N2*	Control	15.93 ± 0.42	22		99
		16.64 ± 0.34	22		101
		14.99 ± 0.19	20		85
	10 μm VK2	17.13 ± 0.54	27	0.003	84
		17.95 ± 0.12	24	<0.001	95
		17.01 ± 0.78	26	0.003	106
	1 μm VK2	17.46 ± 0.55	28	<0.001	93
		17.93 ± 0.19	26	<0.001	114
		18.01 ± 0.25	29	<0.001	104
	0.1 μm VK2	18.88 ± 0.57	29	<0.001	99
		19.34 ± 0.19	31	<0.001	123
		18.75 ± 0.22	28	<0.001	87
	0.01 μm VK2	18.02 ± 0.64	28	<0.001	94
		18.53 ± 0.78	28	<0.001	111
		17.99 ± 0.15	27	<0.001	76
*daf-16(mu86)*	Control	9.01 ± 0.33	17		76
		10.05 ± 0.99	18		93
		9.38 ± 0.36	17		110
	0.1 μm VK2	9.53 ± 0.04	17	n.s.	67
		10.11 ± 0.24	16	n.s.	86
		11.05 ± 0.22	18	n.s.	96
*daf-12(rh286)*	Control	20.14 ± 0.54	31		75
		19.96 ± 0.35	33		79
		20.78 ± 0.17	32		94
	0.1 μm VK2	20.89 ± 0.37	33	n.s.	92
		19.21 ± 0.28	31	n.s.	124
		20.98 ± 0.57	31	n.s.	113
*fat-7(wa36)*	Control	17.64 ± 0.77	25		101
*fat-5(tm420)*		16.98 ± 0.19	24		108
		17.64 ± 0.25	25		94
	0.1 μm VK2	16.62 ± 0.94	26	n.s.	76
		17.14 ± 0.97	25	n.s.	104
		17.27 ± 0.34	25	n.s.	69
*lipl-4 RNAi*	Control	15.46 ± 0.77	21		79
		15.27 ± 0.19	20		88
		16.01 ± 0.35	22		98
	0.1 μm VK2	15.74 ± 0.47	22	n.s.	115
		16.37 ± 0.39	20	n.s.	84
		16.08 ± 0.67	22	n.s.	76

*Mean lifespans were calculated by using Kaplan-Meier method; P-values compared with Controls were determined using the log-rank test; n.s., no significant difference; SEM, standard error of the mean; N, number of worms used*.

Vitamin K has been considered an anti-oxidant in relation to the aging process (35). As such, we tested the function of VK2 on stress resistance in worms. Our results showed that the survival of worms treated with VK2 was significantly improved under heat (37°C) stress (Figure 1C) and H_2_O_2_-induced inner oxidative conditions (Figure 1D).

A recent study also suggested that VK2 might have an effect on immunity (36). *C. elegans* is normally propagated on agar plates seeded with the *Escherichia coli (E. coli*) strain OP50 (28). To address the role of VK2 on immunity in worms, we cultured wild-type worms on a lawn of pathogenic bacteria, *Pseudomonas aeruginosa* (PA14) (*P. aeruginosa*), to test the role of VK2 on resistance to pathogen infection. The results show that N2 worms treated with VK2 demonstrated a significantly enhanced survival compared with control worms (Figure 1E), suggesting that VK2 also influenced the innate immune signaling response.

VK2 levels might impact body fat levels. Interestingly, we found that VK2-treated worms indeed had a significantly lower fat level compared with control worms (Figure 1F). Thus, by using *C. elegans* as a model to study the bioactive effects of VK2 *in vivo*, our results demonstrated that low-dose VK2 treatments have the ability to prolong longevity, increase stress and immunity resistance, and lower body fat content.

### VK2-Extended Lifespan Acts Through DAF-12– and DAF-16–Related Genes

**Figure 2:**
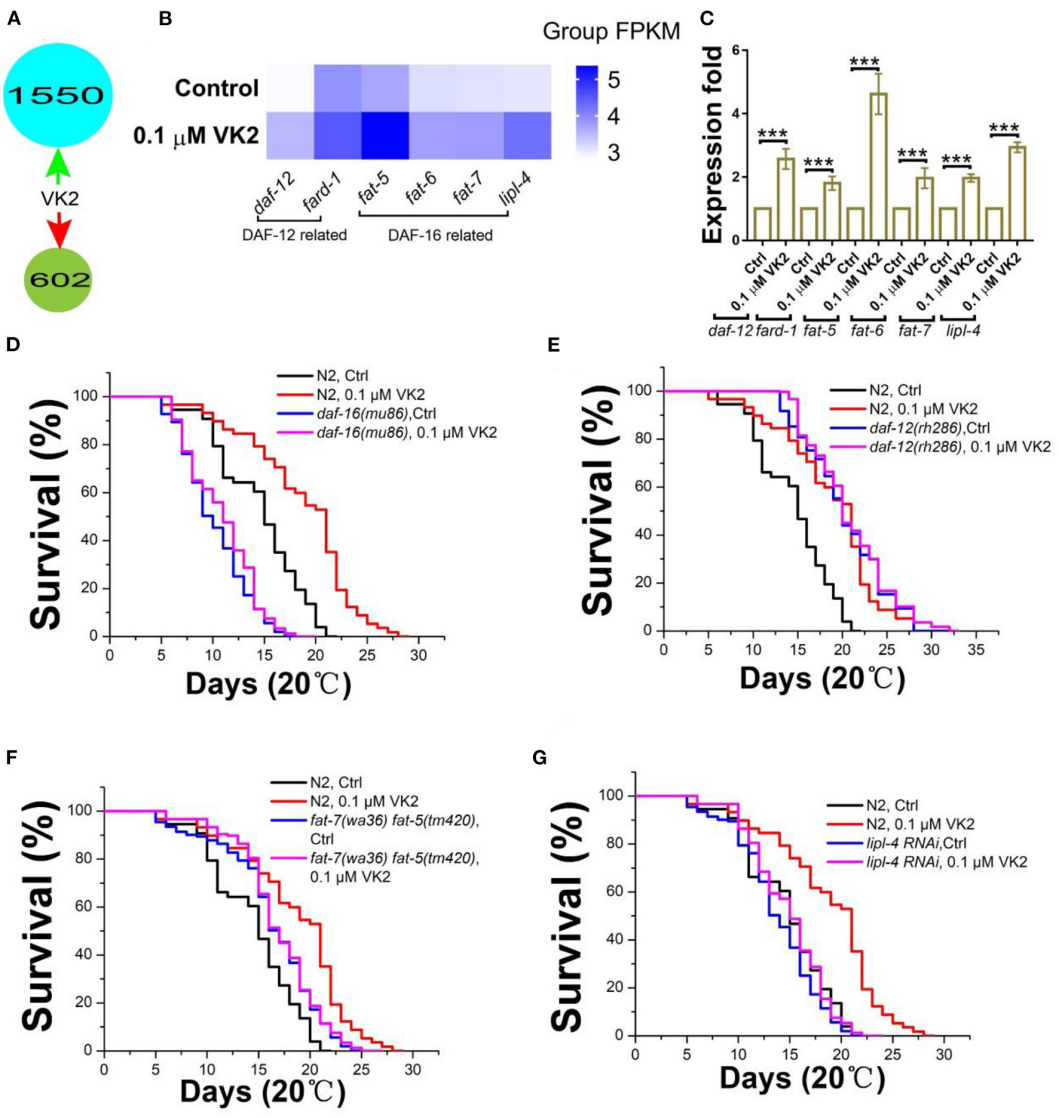
Longevity extension conferred by VK2 treatment in N2 worms is related to the DAF-16/12 target genes. **(A)** RNA-seq data indicate genes up- and down-regulated subsequent to VK2 treatment in N2 worms. **(B)** KEGG pathway enrichment showed that the DAF-16/12-related longevity pathways were affected by VK2 treatment. Six target genes of DAF-16/12 were significantly up-regulated by VK2. The heat-map was made using Group FPKM for both control and VK2-treated worms. **(C)** The expression levels were further confirmed using real-time PCR. ***P < 0.001. **(D)** The lifespan extension conferred by the VK2 treatments was blocked by *daf-16* **(D)** and *daf-12* **(E)** mutants. The results were further confirmed using DAF-12/16 target gene mutants. VK2 failed to significantly extend the lifespan of *fat-5/7* double mutants **(F)**. VK2 extended the lifespan of *lipl-4* RNAi worms, but this was significantly shorter than in control worms **(G)**. The treatments were repeated at least three times, and the sample size was larger than 60 worms in each treatment. The data are summarized in Table 1.

In order to assess how the observed changes in VK2-treated worms were related to gene expression, we performed RNA-sequencing (RNA-seq) analysis to assess differential gene expression patterns. The RNA-seq analysis indicated that 1,550 genes were up-regulated by at least 1.5 times in VK2-treated worms compare with the control, with only 602 genes down-regulated more than 1.5 times (Figure 2A). Pathway enrichment analyses were performed on the up-regulated gene set, and the data showed that six genes, including *daf-12, fard-1, fat-5/6/7*, and *lipl-4*, were enriched among the genes in the longevity pathway in worms. Based on the RNA-seq data, the heat-map analysis indicated that these genes were up-regulated by VK2 treatment (Figure 2B). To confirm the observed changes in gene expression levels, real-time PCR was used to quantify these changes in expression. The results confirmed that all six of these genes were up-regulated in VK2-treated worms compared with the control worms and the expression levels of *fat-5* and *lipl-4* were up-regulated more than the other four genes (Figure 2C). The *fard-1* gene has been reported to be a target gene of DAF-12 (37). As such, the longevity extension function of VK2 was tested on *daf-12* mutants. The results showed that the lifespan extension activity of VK2 was significantly reduced in *daf-12* mutants (Figure 2E). The *daf-16* mutants were treated with VK2, and it was found that the lifespan extension effects of VK2 were significantly diminished in the absence of *daf-16* gene function (Figure 2D). To further confirm that VK2 acted via these genes to extend the lifespan of wild-type worms, the effects on VK2 were tested in *fat-5* mutants and *lipl-4* RNAi worms. The results showed that VK2 had almost no effect on *fat-5/7* mutants (Figure 2F) and that VK2 treatment did not significantly extend the lifespan of *lipl-4* RNAi worms relative to that of control worms (Figure 2G).

### *frk-1* and *lys-7* Play Important Roles in VK2-Related Stress and Immune Responses

**Figure 3:**
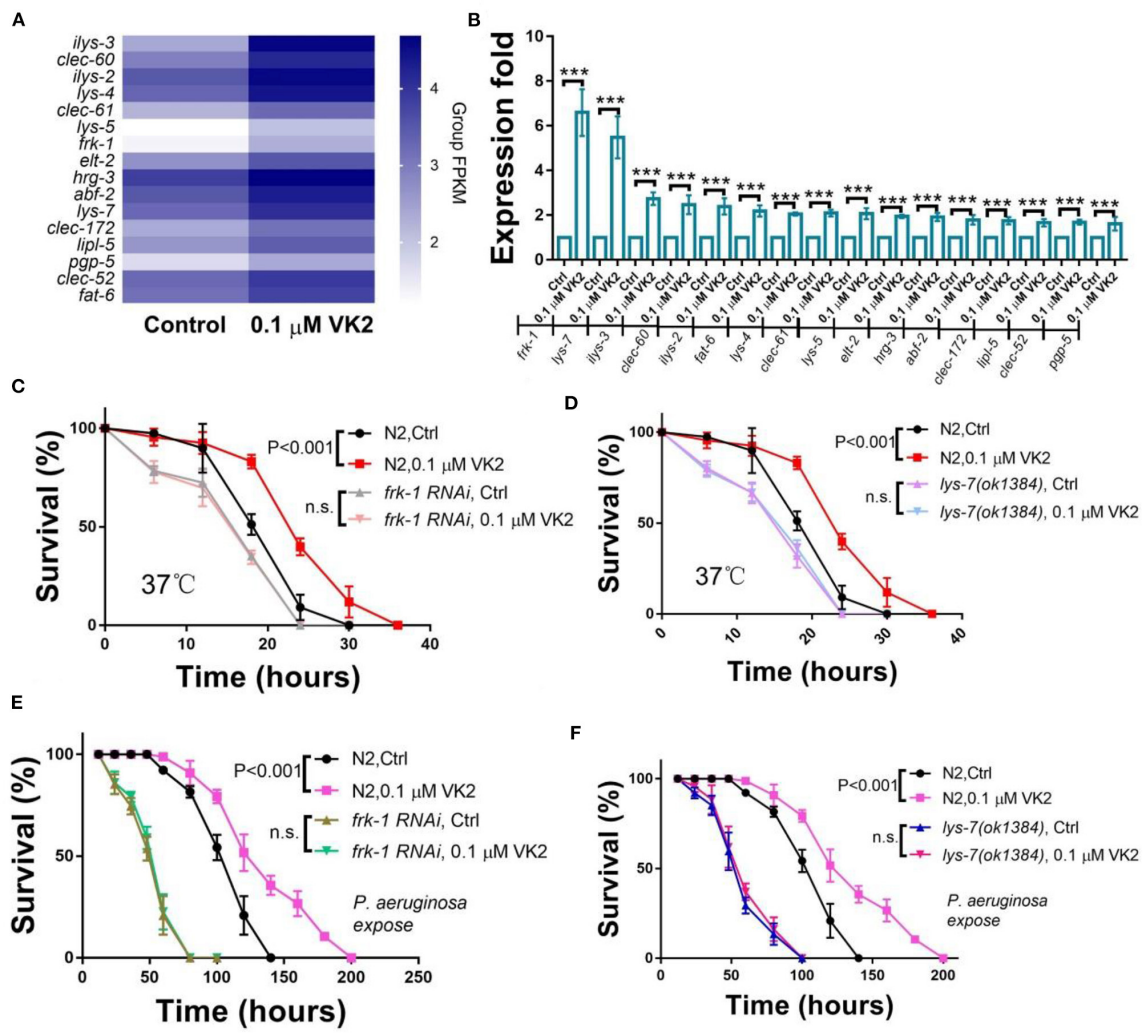
VK2 improves stress resistance mainly through *frk-1* and *lys-7*. **(A)** The biological processes associated with the genes up-regulated by VK2 were analyzed using GO. Sixteen genes were involved in defense response GO terms. Heat-map analysis of these genes was carried out using Group FPKM based on RNA-seq data. **(B)** The expression levels of these 16 genes were further confirmed using real-time PCR. ***P < 0.001. The function of VK2 on survival was absent in *frk-1* RNAi worms **(C)** and *lys-7* mutants **(D)**. Also, VK2 failed to significantly improve the survival of *frk-1* RNAi worms **(E)** and *lys-7* mutants **(F)** cultured with the infective pathogen *P. aeruginosa*. The treatments were repeated at least three times, and the sample size was larger than 60 in each experiment. The *P*-values were determined by using a *t*-test. n.s., no significant difference.

As VK2 can improve the ability of worms to survive in harsh environments, such as those encountered during heat stress and infection, GO enrichment was performed on the set of VK2 up-regulated genes. Forty-five genes were determined to be involved in the biological process of defense and immune response. Among these, 16 genes were identified to have a clear biological function according to the data available on *Wormbase*. The expression levels of these 16 genes affected by VK2 treatment were analyzed via heat-map analysis (Figure 3A) and further confirmed using real-time PCR (Figure 3B). The results showed that all 16 of these genes were up-regulated, with two genes (*frk-1* and *lys-7*) significantly up-regulated by more than five times in VK2-treated worms compared with the control (Figure 3B). *frk-1*, an ortholog of human FER tyrosine kinase, is considered to be involved in several processes, including the innate immune response (38). *lys-7* is also reported to be involved in the defense response in worms (39, 40). To verify that these two genes played important roles in the VK2-improved stress response, we treated *frk-1* knock-down worms and *lys-7* mutants with VK2 under conditions of heat and infection stress. The results showed VK2 failed to significantly improve the survival of *frk-1* RNAi worms (Figure 3C) or *lys-7* mutants (Figure 3D) at 37°C. Additionally, VK2-treated *frk-1* and *lys-7* mutants were cultured on *P. aeruginosa* to test their survival ability. We found that VK2 also failed to significantly improve the survival of *frk-1* RNAi worms (Figure 3E) and *lys-7* mutants (Figure 3F). These results implied that *frk-1* and *lys-7* gene expression were boosted in worms when treated with VK2 to provide protection in these harsh environments.

### Peroxisome Function Is Enhanced by VK2

**Figure 4:**
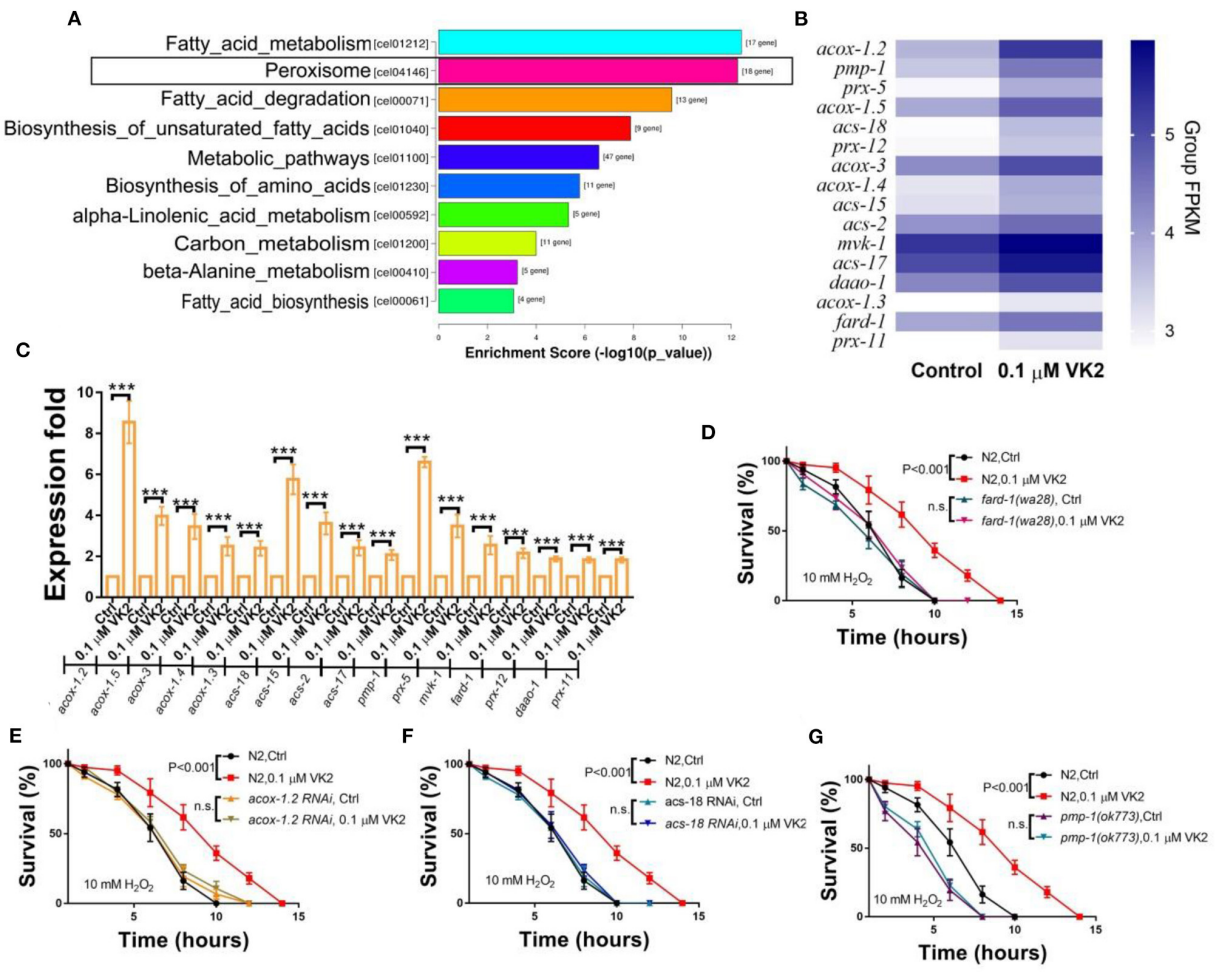
VK2 promotes fatty acid degradation in the peroxisome. **(A)** The second most significantly enriched KEGG pathway among the up-regulated genes induced by VK2 was Peroxisome. There were 18 genes enriched for Peroxisome. **(B)** Heat-map analysis of these genes was carried out using Group FPKM based on RNA-seq data. **(C)** The expression levels of these 18 genes were further confirmed using real-time PCR. ***P < 0.001. *acox-1.2, acs-18*, and *pmp-1* were the top three genes up-regulated by VK2 according to the real-time PCR data. The effects of VK2 on oxidant resistance were blocked by *fard-1* mutants **(D)**, *acox-1.2* RNAi **(E)**, *acs-18* RNAi **(F)**, or *pmp-1* mutations **(G)**. The treatments were repeated at least three times. The *P*-values were determined using a *t*-test. n.s., no significant difference.

The RNA-seq results show that the peroxisome signaling pathway was the second most significantly enriched pathway among the 1,550 genes up-regulated by VK2 (Figure 4A). There were 18 peroxisome-related genes significantly up-regulated, and the expression levels of these genes are shown using a heat-map in Figure 4B. The expression levels of these genes were further confirmed using real-time PCR (Figure 4C). All 18 of these genes were up-regulated by VK2 treatment in wild-type worms, with *acox-1.2, acs-18*, and *pmp-1* up-regulated more than five times compared with the control (Figure 4C). *fard-1* is involved in longevity and is also related to the function of peroxisomes. We cultured *fard-1* mutants under H_2_O_2_-induced (10 mM) oxidative conditions, and the results showed that the VK2 treatment failed to significantly improve the survival of *fard-1* mutants (Figure 4D).

Peroxisomes are oxidative organelles that contain digestive enzymes for breaking down toxic materials in the cell and play an important role in the decomposition of reactive oxygen species (ROS) (41–43). The human orthologs of *acox-1.2* have been implicated in peroxisomal acyl-CoA oxidase activity and are involved in fatty acid β-oxidation (*Wormbase*). Fatty acids are broken down by fatty acid β-oxidation in peroxisomes to produce energy (44, 45). *acs-18* is predicted to activate long-chain fatty acid-CoA ligase, and *pmp-1* is predicted to be a peroxisomal membrane protein and has been shown to enable long-chain fatty acid transporter activity (*Wormbase*). Because all three of these genes are related to fatty acid metabolism via fatty acid β-oxidation in peroxisomes, we tested the effect of VK2 on these mutants. It was found that VK2 treatment failed to improve the oxidative stress resistance induced by H_2_O_2_ in *acox-1.2* RNAi worms (Figure 4E), *acs-18* RNAi worms (Figure 4F), or *pmp-1* mutants (Figure 4G). These results indicated that these three genes play important roles in the fatty acid digestion signaling cascade within peroxisomes and that VK2 can reduce the expression levels of these genes to enhance oxidant resistance.

### VK2 Enhances Fatty Acid Digestion

**Figure 5:**
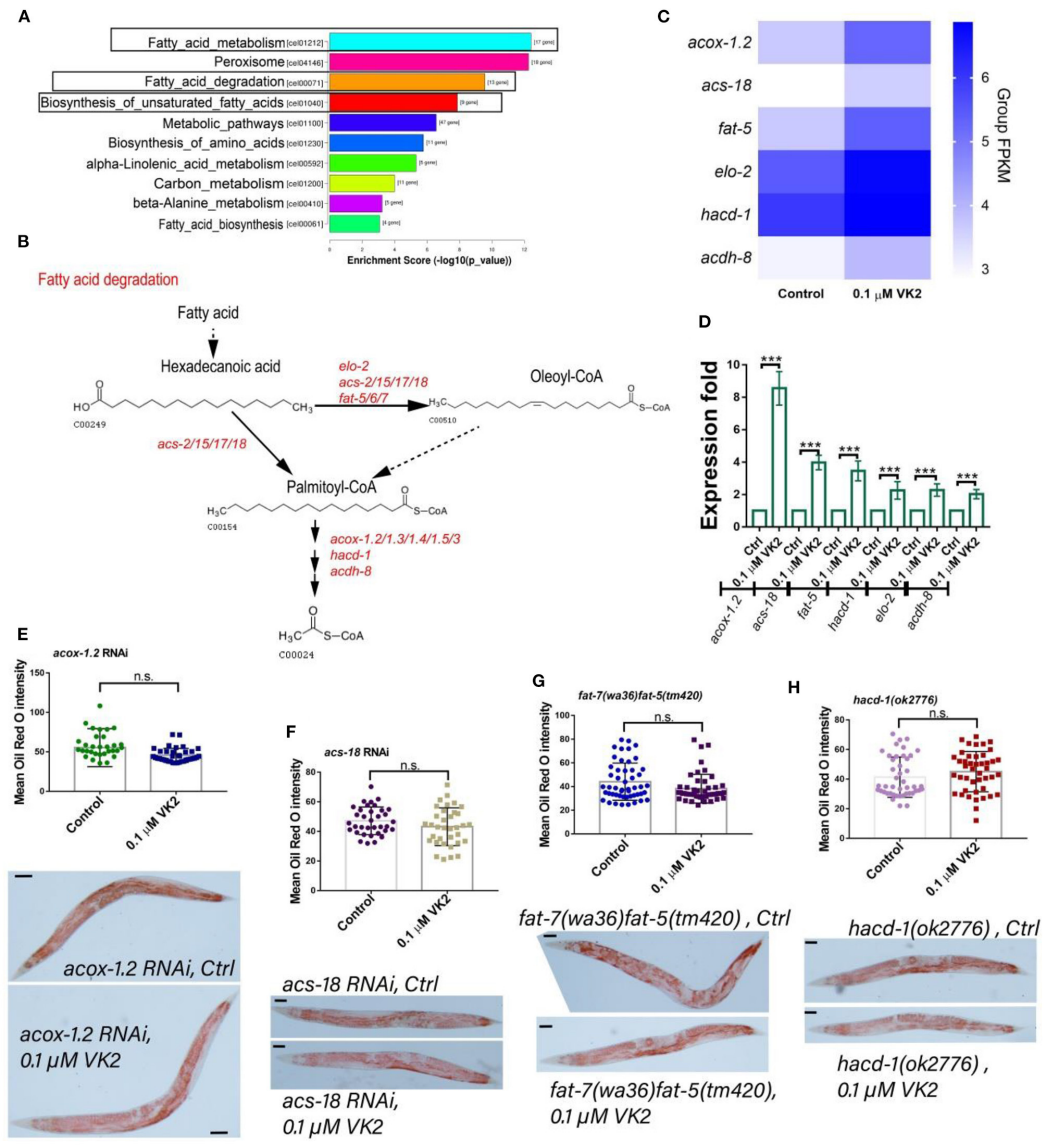
VK2 enhances fat metabolism in worms. **(A)** The most significantly enriched KEGG pathways among the up-regulated genes induced by VK2 were fat metabolism-related pathways: fatty acid metabolism, fatty acid degradation, and biosynthesis of unsaturated fatty acid. **(B)** There were seventeen genes enriched among these pathways, and they each play important roles in these pathways, with their main functions summarized based on KEGG analysis. Fatty acids are elongated, desaturated, and added to CoA by these genes induced by VK2 and then degraded and digested via the process of fatty acid β-oxidation. **(C)** Heat-map analysis of these genes was carried out using Group FPKM based on RNA-seq data. **(D)** The expression levels of these 17 genes were further confirmed using real-time PCR. ***P < 0.001. Among them, *acox-1.2, acs-18, fat-5*, and *hacd-1* were the top four genes up-regulated by VK2 according to the real-time PCR data. The fat content reduction effects of VK2 were blocked by *acox-1.2* RNAi **(E)**, *acs-18* RNAi **(F)**, *fat-5/7* **(G)**, or *hacd-1* mutations **(H)**. The treatments were repeated at least three times, and the sample size was larger than 30 worms in each experiment. Scale bar: 50 μM. The *P*-values were determined by using a *t*-test. n.s., no significant difference.

Based on the above results, it was considered that the longevity, stress resistance, and immune response genes invoked by VK2 treatment almost all function in fatty acid metabolism. More importantly, the RNA-seq results showed that the genes up-regulated by VK2 in worms were significantly enriched in fatty acid metabolism and degradation pathways (Figure 5A). It was speculated that the main function of VK2 in regard to improving the health of the animals was mainly dependent on its ability to enhance fat digestion. To test this hypothesis, we carefully assessed the up-regulated genes induced by VK2 treatment that were enriched in fatty acid related pathways (Figure 5A). It was found that 17 VK2 up-regulated genes were involved in fatty acid metabolism and also played important roles in fat degradation and unsaturated fatty acid biosynthesis (Figure 5A). Among these, 15 genes have defined functions in fat digestion, and their roles in the fat digestion pathway are summarized in Figure 5B. These genes regulate fatty acid elongation and desaturation as well as fatty acid-CoA synthesis. Fatty acid-CoA can be degraded and digested via fatty acid β-oxidation, which is consistent with our findings that VK2 can enhance the fatty acid β-oxidation function of peroxisomes. Interestingly, among them, *acox-1.2, acs-18*, and *fat-5* were confirmed to be involved in regulating longevity (Figures 2B,C) and stress resistance (Figures 4B,C) and also enriched in the fat metabolism pathways (Figures 5C,D). The other three genes related to fat metabolism significantly regulated by VK2 were also analyzed (Figure 5C) and further confirmed using real-time PCR. The results showed that *hacd-1, acdh-8*, and *elo-2* were also significantly up-regulated in VK2-treated worms (Figure 5D). Next, the fat content was assessed in *acox-1.2* RNAi, *acs-18* RNAi, *fat-5*, and *hacd-1* mutants. The results showed that the fat mass in the body of *acox-1.2* RNAi worms (Figure 5E) and *acs-18* RNAi worms (Figure 5F) as well as *fat-5* (Figure 5G)*, hacd-1* (Figure 5H) mutants was not significantly reduced compared to controls following VK2 treatments. These results further confirmed that VK2 could enhance fat digestion mechanisms, especially fatty acid β-oxidation, to improve the survival of worms.

## Discussion

**Figure 6:**
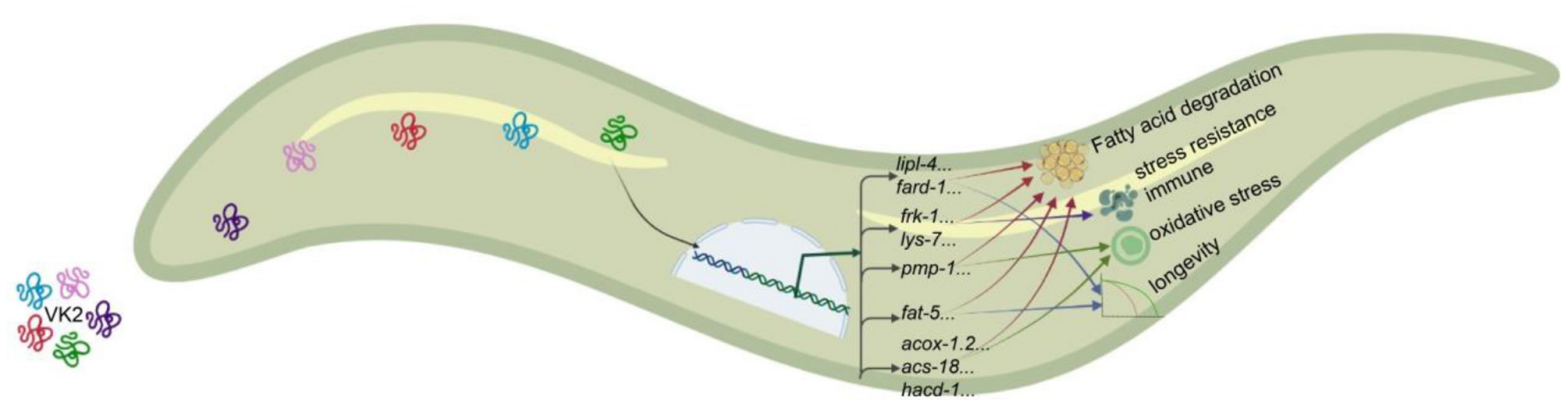
VK2 mainly works via fat metabolism to improve the survival of worms. According to all the data, including RNA-seq and *in vivo* experiments, it was shown that the main function of VK2 involves enhancing fatty acid metabolism. VK2 treatment reduced the fat content in the worm body by activating gene expression involved in longevity, peroxisome, and immune and stress response pathways. However, it was found that almost all of these genes were related to fatty acid metabolism. All of the genes tested in these *in vivo* experiments function in fat metabolism to promote fatty acid digestion, and fatty acid β-oxidation in the peroxisome might play a primary role in VK2-improved fat reduction. As such, the genes induced by VK2 regulate fatty acid elongation, desaturation, and oxidation to promote digestion of fat content and, thus, support the survival of the animal.

VK2 is known to have both antioxidant and anti-inflammatory activities and can influence the aging process. Prior studies have mainly focused on the influence of VK2 on age-related diseases, but most of these consist of case reports or in *vitro* experiments (14, 18, 22). In this report, we performed an *in vivo* study to verify the function of VK2 *in situ* using the *C. elegans* model. Subsequent to VK2 treatment, the entire transcriptional gene profile in VK2-treated worms was comprehensively assessed. This study confirmed that VK2 influences longevity extension, stress resistance, and the immune response in worms. The data analysis showed that the primary function of VK2 might act by regulating fat digestion to improve survival (Figure 6). Considering that the function of VK2 in regard to lipid metabolism, obesity, and diabetes has been gaining more attention in recent studies (7, 23, 24), this current study provides substantial insight into how VK2 regulates fat metabolism, especially fat digestion within the animal body, to ameliorate survival. Together, these findings provide a possible mechanism and support the use of VK2 for the treatment of patients with obesity and/or diabetes.

This study suggested that VK2 mainly confers its effects by enhancing fatty acid digestion within the animal. As the results indicate, VK2 significantly up-regulated the longevity pathway genes *fat-5/6/7, lipl-4*, and *fard-1*. The *fat-5/6/7* and *lipl-4* gene products are involved in the insulin signaling pathway and have been reported to be target genes of the transcription factor DAF-16 (37, 46–48). Together, these results confirmed that VK2 could evoke DAF-12/16 target gene expression to extend lifespan. Interestingly, all six of these genes are related to fat metabolism, suggesting that VK2 might primarily regulate fatty acid metabolism to influence longevity. Though all of these genes are involved in longevity signaling cascades, they also play important roles in controlling fat metabolism. The *fat-5/6/7* genes are stearoyl-CoA 9-desaturases present in worms and serve to introduce double bonds into the fatty acid chains bound to CoA. The long-chain acyl-CoA can then be digested via fatty acid β-oxidation (44, 49). *lipl-4* and *fard-1* have been reported to regulate the lifespan of worms and are also known to function to reduce lipid content (50, 51). As such, the lifespan extension effects of VK2 are likely directly related to its impact on fat metabolism. The antioxidant and anti-inflammatory properties have been previously recognized as the main functions of VK2 based on its protective action by way of limiting the accumulation of intracellular free radicals, thus reducing reactive oxygen species, and the inhibition of nuclear factor kappa B (NF-κB) activation, thus decreasing the production of pro-inflammatory cytokines (52–54). However, in this study, we showed that VK2 can influence gene function affecting the peroxisome and immune system. The human FER tyrosine kinase ortholog gene, *frk-1*, and a predicated lysozyme gene, *lys-7* (38–40), were significantly up-regulated manyfold following VK2 treatment, both of which are considered involved in regulation of the innate immune and stress defense responses. Interestingly, increasing the expression of both these genes was previously reported to result in decreasing fat content (55, 56). This suggests that VK2 might also influence genes related to fat metabolism resulting in an improved immune and stress response in worms. Recent studies also imply that VK2 can regulate cell differentiation and proliferation (18, 20), and this might be due to its influence on *frk-1*, an oncogene-related kinase. The expression levels of the peroxisomal genes *acox-1.2, pmp-1*, and *acs-18* were found to be significantly increased following VK2 treatment. *acs-18* is a long-chain fatty acid-CoA ligase, which adds the -CoA onto the fat chains, allowing fatty acid-CoA to be digested by fatty acid β-oxidation in the peroxisome. *pmp-1* is a peroxisomal membrane protein that can transport long-chain fatty acids into the peroxisome, while *acox-1.2* is an acyl-CoA oxidase that enables fatty acid β-oxidation to complete fat digestion. Increased levels of fatty acids broken down by fatty acid β-oxidation in the peroxisome to generate energy might result in less fatty acid processed in the mitochondria, thus limiting the production of oxidative agents. These results suggest that VK2 could improve fatty acid digestion in the peroxisome to enhance the ability of worms to defend against oxidative stress. Together, these observations support the idea that fatty acid digestion occurring in different organelles might be influenced by VK2, with an overall result of improving the health and stress resistance of the animal.

Finally, our RNA-seq and *in vivo* experiments together confirmed that VK2 can significantly impact fat digestion to support the animal's survival. The top signaling pathways affected by VK2 treatment were mostly those related to fatty acid digestion. Interestingly, the genes controlling fatty acid digestion up-regulated by VK2 are known to be involved in regulating longevity, stress defense, and fatty acid β-oxidation in peroxisomes. This is especially true of the most strongly increased genes induced by VK2: *acox-1.2, fat-5*, and *acs-18*, which play key roles in fatty acid digestion, longevity extension, and stress defense. Overall, our results support the role of VK2 in enhancing fatty acid digestion to improve the general survival ability of worms, and this novel mechanism of action redefines our understanding of the bioactivity of VK2.

These findings regarding the function of VK2 on fat metabolism should promote additional studies on the role of VK2 and support its use in treating patients with obesity and/or diabetes. According to a recent study, about 20% of adults worldwide will present with obesity by 2025 (4), and hospitalized patients with COVID-19 were three times more likely to die of the infection if they had a combination of metabolic disorders, such as obesity and diabetes (57).

The gut microbiota functionally influences host genetic events primarily through specific media molecules produced by the bacteria. As a fat-soluble molecule, VK2 is mainly produced by the intestinal microbiota and might function as a media molecular to promote the communication between the host and intestinal microbiota. Considering that our study showed VK2 mainly works on fatty acid digestion in animals, VK2 could be used as a supplement to treat obesity and diabetes. However, the role of VK2 as a molecular messenger produced by the intestinal microbiota to influence the host heath and if it can be used clinically to treat obesity and diabetes still requires further investigation to support the use of VK2 to improve human health.

## Conclusion

Our study suggested that fatty acids are elongated, desaturated, and then synthesized to fatty acid-CoA by the genes influenced by VK2 treatment in worms, with fatty acid-CoA likely degraded and broken down via fatty acid β-oxidation into acyl-CoA. These findings implied that VK2 can enhance fat metabolism, especially fatty acid β-oxidation–related degradation, to reduce the fat contents in the animal body, thus improving the survival and health of the animal.

## Data Availability Statement

The datasets presented in this study can be found in online repositories. The names of the repository/repositories and accession number(s) can be found at: GEO Database with accession GSE199145.

## Author Contributions

ZQ and SZ contributed to conception, design of the study, performed the statistical analysis, and wrote sections of the manuscript. WH, LZ, and ZQ performed the experiments. SZ organized the database and wrote the first draft of the manuscript. All authors contributed to manuscript revision, read, and approved the submitted version.

## Funding

The work was supported by grants from the Henan University (Yellow River Scholar Fund) and Key Scientific Research Project Plan of Henan Province (Grant No. 22A310011 and 21A330001), Science and Technology Development Plan of Kaifeng in 2021 (Grant No. 2103007), and Henan Province's key R&D and promotion projects (scientific and technological research) projects (Grant No. 222102310587).

## Conflict of Interest

The authors declare that the research was conducted in the absence of any commercial or financial relationships that could be construed as a potential conflict of interest.

## Publisher's Note

All claims expressed in this article are solely those of the authors and do not necessarily represent those of their affiliated organizations, or those of the publisher, the editors and the reviewers. Any product that may be evaluated in this article, or claim that may be made by its manufacturer, is not guaranteed or endorsed by the publisher.
