# Supplementary material for: Vitamin K2 Enhances Fat Degradation to Improve the Survival of C. elegans
Source: Front Nutr. 2022 Apr 15;9:858481. doi: 10.3389/fnut.2022.858481 (PMC9051363; doi:10.3389/fnut.2022.858481)
Supplement: Supplementary file 1 [file Table_1.docx]

Table S1. Primers for real- time PCR.

| gene | forward | reverse |  |  |
| --- | --- | --- | --- | --- |
| *ilys-3* | TAATTTCAGCGGTCGCCTAC | GGATCCGACGTCCATGTTAC | | |
| *clec-60* | GTTTTCCGTGCTCTTTCGTC | ACGGTTGTACCCTTGTTTGC | | |
| *ilys-2* | CTGGAAGAGATGTGCTGACG | CCCACTACCTTGACGGCTTA | | |
| *lys-4* | GAGGCAATGGTCAGAGAAGC | ATCATAGGTTGCCCACCAGA | | |
| *clec-61* | CACATCAATCGACCAACTCG | ATCATCAGCTGCCTCCAATC | | |
| *lys-5* | AGGCGTCTCTTGATGCAGTT | CTTCAATGAATGCACGGTTG | | |
| *frk-1* | ATGAAAATGCCAGACGGAAC | TCTCGGAGCTTCTTGTGGAT | | |
| *elt-2* | AAAGCGGAGACTCTTCCACA | GCCGGTTAGATGACCTTTGA | | |
| *hrg-3* | TCGGCCGGTAAGTTTTATCA | TTTTGGACGGAAAAGATGATG | | |
| *abf-2* | TGCCAGAATGGATGTTCCTA | CCGGTAACACACACAAGTCG | | |
| *lys-7* | TGGAGCAACATTGGAAATGA | GAGCTGTCCAGCATCCAAAT | | |
| *clec-172* | ATGCTATTACCCGGCAAGTG | TCCAGGAAAAACTCGGAAAA | | |
| *lipl-5* | CATGTGTTGGAGGTCACTGG | CTTCGCGAAACTTCCATCAT | | |
| *pgp-5* | CTCTTGGGGCAAAATGAAAA | CAACTTCTTGCTGCTGTCCA | | |
| *clec-52* | CCATCTGTGCCACATTGAAC | GCTCCACCGTCAATGAATTT | | |
| *fat-6* | GTGATCATCGTTGCCATCAC | GTGGATGCTTACGCACAAGA | | |
| *acox-1.2* | TTTTCGTCGTTTTCCCTTTG | TTGCACTTTCCTGCTGATTG | | |
| *pmp-1* | ATCGTTGGATGGGTAGCTTG | TTCCGACAATTTCAGCATCA | | |
| *prx-5* | GTTTCAACCGCGACAGGTAT | AGATTCCATGCTTGGAGCAC | | |
| *acox-1.5* | TAACGGATTTTTGGCCTTTG | GAGATGGAGCCGTGTAGAGC | | |
| *acs-18* | TGTGGCTCCAGATCTCACAG | CTGGATCTGGGACAACGATT | | |
| *prx-12* | ACCGGCTTTACAGCATCTTG | CGGTGAACCGAAAGAAATGT | | |
| *acox-3* | TTTTAGCTCACTCGGCCACT | TGGAAAGTTCTAGGGCAACG | | |
| *acox-1.4* | TCAGAGAGGTTACGGGGATG | AGCTCTCCCCAACCATTTTT | | |
| *acs-15* | CGATATTGCGGAACTTGGAT | ATGCTGATGCAGTTGCTTTG | | |
| *acs-2* | TGACGTGCTCAAGTCTCCAC | CTTCACCATCTTCTCGCACA | | |
| *mvk-1* | GACGTCTTCTCTGCGTTTCC | CCACCCAATTTCTTTGCCTA | | |
| *acs-17* | CGGAGAGAGTCAAGGTACGC | CACGTTCTTCCATGGTGTTG | | |
| *daao-1* | GGTACCTGGAACCAACTCCA | CATAGCTTCTTGCCACGTCA | | |
| *acox-1.3* | CATGAGCTTGCAGAATCCAA | CTGTTGAACGGGGTCTGATT | | |
| *fard-1* | CCGAGACAATGTGAGGGAGT | GATGCTCGTCGAACTTGACA | | |
| *prx-11* | TGACGCTTGTCGGATTATCA | ATGAAAACGAGGCACTCTGC | | |
| *daf-12* | GGTTTCCCAAAAATCCCAGT | ACGAAACGCCTCATGGTAAC | | |
| *fat-5* | AGTGCAAGAAGTTCGGCTGT | CAGAAACACGCCAGAGATCA | | |
| *fat-7* | GTGATCATCGTTGCCATCAC | GTGGATGCTTACGCACAAGA | | |
| *lipl-4* | GCCGAGAAGTTCCTACATCG | CCAGCACTTTGATGAGGTGA | | |
| *elo-2* | TGTGATGTCGAAAGCTCCAG | CAAATGTGAGGGCATGATGA | | |
| *hacd-1* | TCATTGTCAACCGGCTATTG | AGAAGTTCCAAATCGCATGG | | |
| *acdh-8* | GCTGGATTCAAAGTGGCAAT | TGCATGTCTTTCCATTGCAT | | |
| *cdc-42* | CTGCTGGACAGGAAGATTACG | CTCGGACATTCTCGAATGAAG | | |
